# Experiential Learning in Refugee Health: A Convergent Parallel Mixed-Methods Study of Medical Student Challenges and Educational Needs in a Student-Run Clinic

**DOI:** 10.7759/cureus.113166

**Published:** 2026-07-22

**Authors:** Morad Marikh, Omar Alkhabbaz, Saad Sadaf, Zaid Qaddura, Bilal Siddiqi, Raheela Hafeez

**Affiliations:** 1 Paul L. Foster School of Medicine, Texas Tech University Health Sciences Center El Paso, El Paso, USA; 2 Texas College of Osteopathic Medicine, University of North Texas Health Science Center, Fort Worth, USA; 3 School of Medicine, Sam Houston State University College of Osteopathic Medicine, Conroe, USA; 4 Department of Pediatrics and Women's Health, University of North Texas Health Science Center, Fort Worth, USA

**Keywords:** convergent parallel mixed methods, cultural competency, experiential learning, language barriers, medical education, refugee health, student-run clinic

## Abstract

Background: Refugees face compounding health burdens and access barriers that require culturally responsive, linguistically adapted clinical care. Student-run refugee health clinics offer high-value training environments but are rarely studied as sites of structured experiential learning. Grounded in Kolb's Experiential Learning Theory (ELT), this study examined where the learning cycle breaks down for medical students in a community refugee clinic, with a focus on language barriers, cultural challenges, and resource limitations as the primary disruptions to effective learning and care.

Methods: A cross-sectional, convergent parallel mixed-methods survey was administered to medical students (n = 22) who completed at least one clinical session at a biweekly refugee health clinic in the Dallas-Fort Worth (DFW) metroplex over the preceding 12 months, with quantitative and qualitative data collected concurrently and integrated during interpretation. The 22-item instrument used Likert-scale, multiple-choice, and open-ended items organized around three domains derived from Kolb's ELT. Quantitative data were analyzed using descriptive statistics, Mann-Whitney U tests, and Spearman correlations. Open-ended responses underwent directed content analysis by two independent coders using a deductive framework derived from Kolb's ELT stages.

Results: Language discordance was ubiquitous; 90.9% (20/22) of participants reported language barriers often or always, yet 72.7% (16/22) reported inadequate formal training in interpreter use. Cultural challenges were frequent (95.5% (21/22) often or sometimes) and were associated with uncertainty navigating religious norms and gender sensitivities. Perceived faculty and peer support showed a negative correlation with reported resource limitations (rho = -0.425, p = 0.049); given the small sample size, this finding should be interpreted cautiously and viewed as hypothesis-generating rather than confirmatory. Directed content analysis identified four themes: interpreter inaccessibility, cultural and gender-concordant care gaps, inadequate pre-clinic preparation, and structural workflow deficits.

Conclusions: Medical students encounter substantial structured learning disruptions in refugee clinic settings that are amenable to intervention. Applying Kolb's ELT as an analytic framework suggests that deficits may occur more at the reflective and conceptual phases than at the level of direct experience, though this interpretation is drawn from survey and thematic data rather than direct measurement of each stage. These findings suggest that structured interpreter training, simulation-based cultural communication curricula, and intentional faculty mentorship may inform future educational interventions aimed at improving both student learning and equitable refugee care delivery.

## Introduction

Globally, there were more than 117.3 million forcibly displaced persons as of the end of 2023, a record high driven by conflict, climate displacement, and political persecution [1]. Within the United States, refugees and asylum seekers represent a growing patient population whose health needs are disproportionate and complex: higher burdens of infectious disease, untreated chronic conditions, nutritional deficiencies, and trauma-related mental health disorders compounded by structural barriers including language discordance, cultural incongruence, unfamiliarity with Western medical systems, and insurance gaps [1,2]. Delivering equitable care to this population demands clinical competencies, multilingual communication, trauma-informed interviewing, and cross-cultural negotiation that are rarely systematically taught in standard medical curricula [3].

Student-run refugee health clinics have emerged as a pragmatic response to both the service gap and the educational need. These clinics provide accessible, low-barrier care in community settings while simultaneously positioning medical students as primary providers under faculty supervision. The experiential richness of this model - complex underserved patients, limited resources, and authentic clinical decisions - makes it a theoretically ideal training environment. Yet this potential is rarely formalized. Without structured educational scaffolding, students may leave clinical sessions having accumulated exposure but without translating that exposure into transferable competence.

Several educational frameworks could inform analysis of this gap, including Situated Learning Theory, Communities of Practice, and Transformative Learning [4-6]. We selected Kolb's Experiential Learning Theory (ELT) because its explicit four-stage cycle allows barriers to be mapped not only to whether learning occurs but to where in the learning process it breaks down, a level of diagnostic specificity that is central to our research question [7]. ELT posits that learning proceeds through a four-stage cycle: (i) Concrete Experience, direct engagement with a situation; (ii) Reflective Observation, structured consideration of what occurred; (iii) Abstract Conceptualization, formation of generalizable principles; and (iv) Active Experimentation, deliberate application of those principles in new contexts. ELT has been extensively applied in health professions education, particularly to understand what conditions allow clinical experience to mature into competence [8,9]. Crucially, ELT predicts that absent structured opportunities for reflection and conceptualization, concrete experience, however intense, will not reliably produce learning. This makes it particularly well-suited to analyzing refugee clinic training, where students routinely confront novel challenges without formal debriefing or scaffolded conceptualization.

Prior studies have documented medical student underpreparedness for refugee and immigrant health care in general outpatient settings [10]. However, while prior work has documented general challenges in refugee and immigrant health education, few studies have specifically examined the clinician-level barriers students encounter within student-run refugee clinic models or systematically analyzed how those barriers disrupt the experiential learning cycle using an established educational framework. Understanding which stages of Kolb's cycle are most disrupted and why is essential for designing targeted, theory-grounded educational interventions.

This study addresses that gap. Using a mixed-methods approach grounded in Kolb's ELT, we investigated the challenges medical students encounter while volunteering at a biweekly refugee health clinic in the Dallas-Fort Worth (DFW) metroplex. Our primary objective was to determine where, within Kolb's experiential learning cycle, structural and educational barriers most disrupt student learning in a refugee clinic setting. Secondary objectives were to: (i) characterize the frequency and nature of language, cultural, and resource barriers; (ii) assess student preparedness and perceived support; and (iii) identify student-proposed educational solutions. By grounding both our analysis and our recommendations in ELT, we aimed to provide educators with a theoretically coherent framework for redesigning refugee clinic training.

## Materials and methods

This study employed a cross-sectional, convergent parallel mixed-methods design combining quantitative survey analysis with directed qualitative content analysis. The setting was a student-run refugee health clinic operating on a biweekly schedule at a faith-based community organization serving newly resettled refugees in the DFW metroplex. The clinic primarily serves Arabic-, Swahili-, Burmese-, and Spanish-speaking patients from East African, Middle Eastern, and Southeast Asian communities. Each session includes a pre-clinic briefing, student-led history-taking and examination under faculty supervision, interpreter-assisted encounters when available, and post-clinic discussion. The clinic is affiliated with two osteopathic and allopathic medical schools, drawing students from all training years.

Conceptual framework

Kolb's ELT was used both as an organizing framework for survey development and as an analytic lens for interpreting findings [7]. Each of the three primary barrier domains (language, culture, resources) was mapped to ELT stages: Concrete Experience (barrier frequency and impact on clinical encounters), Reflective Observation (preparedness, confidence, perceived support), and Abstract Conceptualization/Active Experimentation (proposed educational solutions). This framework operationalized ELT beyond a rhetorical reference; it directly guided item construction, domain grouping, and interpretive analysis (Table 1).

**Table 1:** Alignment of Kolb’s ELT stages with survey domains and representative items ELT: Experiential Learning Theory

Kolb's ELT stage	Definition	Survey domain mapped	Representative survey items
Concrete Experience	Direct engagement with clinical situation	Language barriers; cultural challenges; resource limitations	"How frequently do you encounter language barriers?" / "How often do cultural differences affect your clinical encounter?"
Reflective Observation	Structured consideration of experience	Interpreter confidence; cultural preparedness; perceived support	"Rate your confidence using interpreters." / "Do you feel faculty/peer support is adequate?"
Abstract Conceptualization	Formation of generalizable principles	Training adequacy; proposed curricular improvements	"Was your prior training adequate to address language barriers?" / "What training would have been most helpful?"
Active Experimentation	Application of principles in new contexts	Structural/workflow recommendations	"What structural changes would improve patient care and student learning at this clinic?"

Mixed-methods integration

This study used a convergent parallel mixed-methods design, reported in accordance with the Good Reporting of a Mixed Methods Study (GRAMMS) framework [11]. Quantitative and qualitative data were collected concurrently, analyzed separately using their respective methods, and integrated during the interpretation phase. Integration was structured around Kolb's ELT stages: quantitative findings characterized the frequency and distribution of barriers, while qualitative themes provided explanatory depth for those quantitative patterns. Points of convergence and divergence between the two strands were explicitly assessed during interpretation and are highlighted in the discussion.

Participants and eligibility

Eligibility required current enrollment in a Doctor of Medicine (MD) or Doctor of Osteopathic Medicine (DO) program and completion of at least one four-hour clinical session at the refugee clinic within the 12 months preceding survey distribution. Students in all training years (pre-clinical Medical Student Year 1 - Medical Student Year 2 (MS1-MS2) and clinical Medical Student Year 3 - Medical Student Year 4 (MS3-MS4)) were eligible. There were no exclusions by gender, age, language background, or prior refugee health exposure. This intentionally inclusive approach captured the full heterogeneity of student perspectives across training levels. No a priori sample size calculation was performed. Given the exploratory, pilot nature of this study and the small, well-defined eligible population (approximately 30 students), we aimed for a census sample of all eligible students rather than a pre-specified target sample size; the adequacy of the resulting sample (n = 22) for statistical inference is discussed as a limitation.

Survey instrument development

A 22-item survey instrument (see Appendix A) was developed de novo following a structured development process: first, a systematic review of existing instruments for immigrant/refugee health preparedness [10,12]; second, expert review by three physician educators with expertise in refugee medicine and health professions education; third, alignment of items with Kolb's ELT framework (Table 1); and fourth, cognitive pilot testing with four medical students not participating in the study, resulting in minor wording revisions for clarity. The instrument was not submitted for formal psychometric validation given the exploratory, single-site nature of this pilot study, a limitation explicitly acknowledged.

The survey comprised four sections. Section 1 (demographics) collected training year, number of clinic sessions attended, and language proficiency relevant to the patient population. Section 2 (language barriers and interpreter use) assessed barrier frequency on a five-point Likert scale (1 = never, 5 = always), interpreter confidence, adequacy of prior language training, and strategies used when interpreters were unavailable. Section 3 (cultural challenges and resource limitations) assessed frequency of cultural barriers affecting history-taking and physical examination, situational uncertainty in religious and gender-sensitive contexts, resource availability, and time adequacy. Section 4 (perceived support and training) assessed faculty and peer support, pre-clinic training effectiveness, and proposed solutions through both structured items and open-ended prompts. Three open-ended items asked students to describe their most challenging clinical encounter, what additional training would have been most helpful, and what structural improvements they would recommend.

Recruitment and data collection

The survey was administered electronically via an anonymous online survey platform (Google Forms; Google LLC, Mountain View, USA) with no identifying information collected. Clinic coordinators distributed a study invitation email to all clinic members meeting eligibility criteria. Two reminder emails were sent at two-week intervals. The survey was active for four weeks in the spring academic semester. Participation was anonymous; no personally identifiable information was collected. The response rate was 22 of an estimated 30 eligible students (73.3%).

Ethical considerations

This study was reviewed by the North Texas Institutional Review Board (IRB) (Protocol #2023-187) and granted exempt status under 45 Code of Federal Regulations (CFR) §46.104(d)(2) on May 15, 2025, applicable to research involving educational surveys with minimal risk where responses are recorded in a manner that precludes identification of participants. Anonymous electronic data collection, password-protected storage, and aggregate-only reporting ensured participant confidentiality. Written informed consent was waived consistent with the IRB exemption. Participants viewed a plain-language description of the study before proceeding; continuation constituted implied consent under the approved protocol. No incentives were offered.

Quantitative analysis

Quantitative responses were analyzed using IBM SPSS Statistics for Windows, Version 27.0 (IBM Corp., Armonk, USA) [13]. Descriptive statistics (frequencies, percentages, medians, interquartile ranges (IQRs)) summarized participant characteristics and responses across all Likert-scale and multiple-choice items. Normality was not assumed given the small sample and ordinal data; non-parametric methods were used throughout. Mann-Whitney U tests compared pre-clinical (MS1-MS2) and clinical (MS3-MS4) students on primary outcome variables. Spearman's rank correlation (rho) examined associations between perceived support, preparedness, and barrier domains. Given the exploratory nature of the study and small sample size, statistical findings are presented as hypothesis-generating rather than confirmatory. Significance was set at p < 0.05. Given the small sample size, we report exact p-values throughout, along with effect size estimates (Spearman's rho) where applicable, to allow readers to assess the magnitude and precision of associations rather than relying on significance thresholds alone. No correction for multiple comparisons was applied across the several Mann-Whitney U tests and Spearman correlations performed; given the number of statistical tests conducted, the risk of false-positive findings is acknowledged as a limitation, and all statistical results should be interpreted as exploratory rather than confirmatory.

Qualitative analysis

Open-ended responses were analyzed using directed content analysis [14], in which Kolb's four ELT stages served as a deductive coding framework. Two authors (OA, MM) independently coded all responses. Initial codes were mapped to ELT stages; codes not fitting predefined categories were noted and considered for emergent themes. Discrepancies were resolved through discussion and consensus. A third author (RH, faculty) reviewed a random 30% (7/22 participants' responses) sample for credibility. Inter-rater agreement was assessed using Cohen's kappa prior to consensus (κ = 0.78, indicating substantial agreement). Final themes were synthesized across ELT stages and integrated with quantitative findings to produce a convergent mixed-methods interpretation.

## Results

Participant characteristics

Twenty-two students completed the survey (estimated response rate: 73.3%). Most participants were aged 25-30 years (15/22, 68.2%) and identified as male (14/22, 63.6%). Pre-clinical students (MS1-MS2) comprised 54.5% (12/22) of the sample; clinical students (MS3-MS4) comprised 45.5% (10/22). The majority (13/22, 59.1%) had attended 1-3 clinic sessions; 22.7% (5/22) had attended more than six sessions. Half of respondents spoke a language commonly used by the patient population (11/22, 50.0%), while 36.4% (8/22) reported no relevant language proficiency, and 13.6% (3/22) reported only basic familiarity (Table 2).

**Table 2:** Participant demographic characteristics (n = 22) MS1-MS2: Medical Student Year 1 - Medical Student Year 2; MS3-MS4: Medical Student Year 3 - Medical Student Year 4

Characteristic	n (%)
Age (years)	
25-30	15 (68.2)
>30	7 (31.8)
Gender	
Male	14 (63.6)
Female	8 (36.4)
Training level	
Pre-clinical (MS1-MS2)	12 (54.5)
Clinical (MS3-MS4)	10 (45.5)
Clinic sessions attended	
1-3	13 (59.1)
4-6	4 (18.2)
>6	5 (22.7)
Language proficiency (patient population language)	
Proficient/fluent	11 (50.0)
Basic familiarity	3 (13.6)
None	8 (36.4)

Concrete Experience stage - frequency and nature of barriers

Language barriers were nearly universal. Around 20 of 22 students (90.9%) reported encountering language barriers often or always (median 4.0, IQR 4.0-5.0). Interpreter confidence was moderate (median 3.0, IQR 2.0-3.75), and 72.7% (16/22) of students reported inadequate formal training in managing language discordance. When interpreters were unavailable, students most commonly relied on bilingual companions of the patient (13/22, 59.1%) or basic phrase lists (10/22, 45.5%); only 27.3% (6/22) reported access to on-demand professional telephonic interpretation.

Cultural differences affected clinical encounters frequently: 50.0% (11/22) reported impact often and 45.5% (10/22) sometimes (median 3.5, IQR 3.0-4.0). The most common source of uncertainty was navigating religious observance during physical examination (reported by 14/22, 63.6%) and ensuring appropriate gender concordance during sensitive examinations (12/22, 54.5%). Resource limitations were more variable; most students reported constraints rarely or sometimes (median 3.0, IQR 2.0-3.0). However, 40.9% (9/22) reported time limitations that constrained their clinical interactions.

Reflective Observation stage - preparedness, confidence, and support

Despite high barrier frequency, most students rated their overall cultural preparedness as adequate (median 4.0, IQR 3.0-4.0). However, this perception of preparedness did not correlate with cultural barrier frequency (rho = 0.18, p = 0.42).

**Figure 1:**
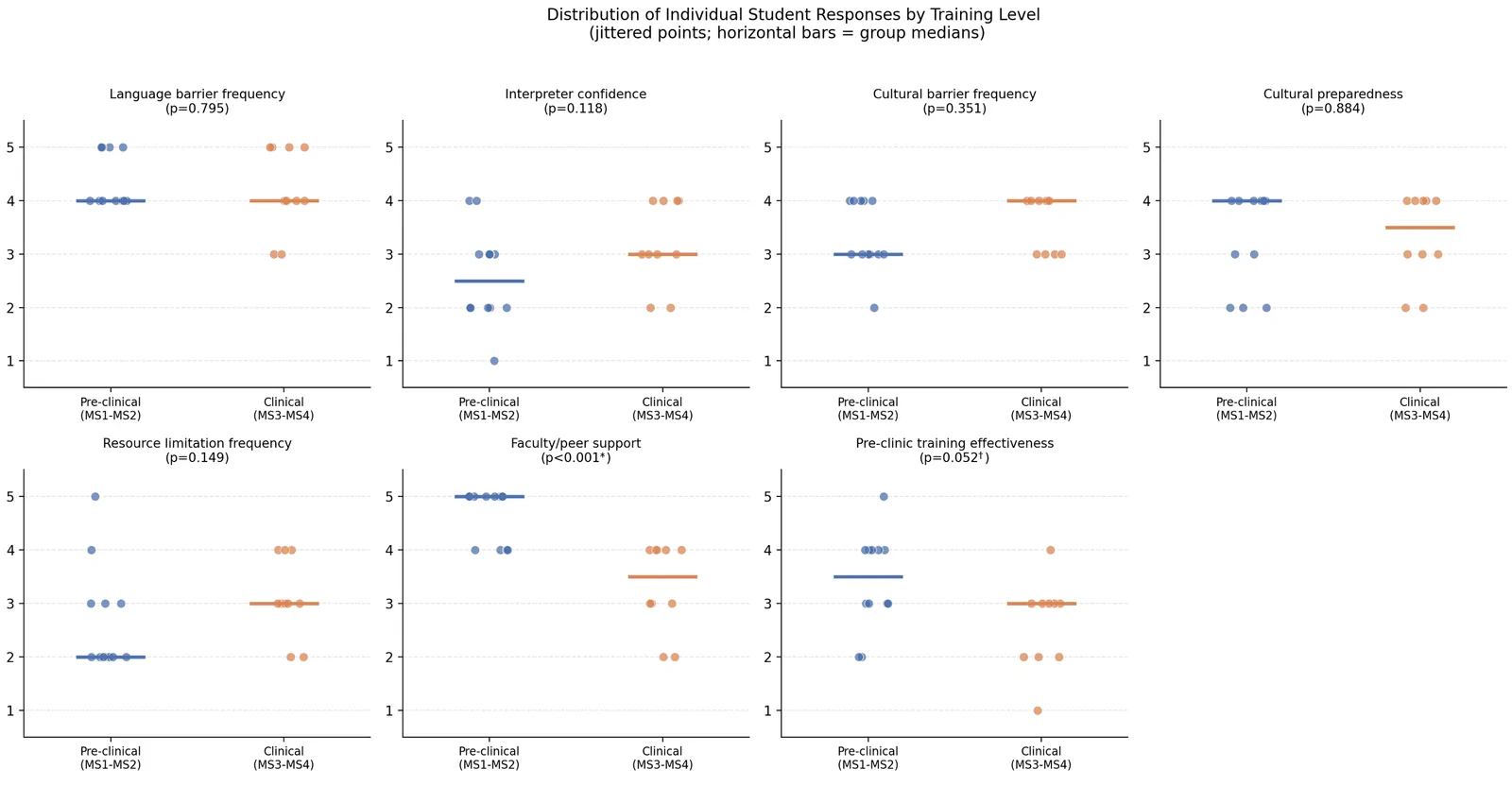
Distribution of individual student responses by training level *p < 0.05; ^†^approaching significance (p < 0.10). All p-values are exploratory (Mann-Whitney U). Likert scale: 1 = never/not at all, 5 = always/very much. MS1-MS2: Medical Student Year 1 - Medical Student Year 2; MS3-MS4: Medical Student Year 3 - Medical Student Year 4

Perceived faculty and peer support was generally high (median 4.0, IQR 4.0-5.0). Pre-clinical students reported significantly higher support levels than clinical students (median 5.0 vs. 3.5, Mann-Whitney U p = 0.0005), a difference that may reflect differences in supervised engagement or faculty investment across training levels (Figure 1 and Table 3, which summarize median, IQR, and Mann-Whitney U comparisons by training level for all primary quantitative outcomes). Higher perceived support was negatively correlated with reported resource limitations (rho = -0.425, p = 0.049); given the sample size and the proximity of this p-value to the conventional threshold, this association should be interpreted with caution and considered exploratory rather than a definitive relationship.

**Table 3:** Summary of quantitative outcomes by training level *p<0.05; ^†^approaching significance (p<0.10). All p-values are exploratory. Likert scale: 1 = never/not at all, 5 = always/very much. IQR: interquartile range

Outcome	Pre-clinical median (IQR)	Clinical median	Mann-Whitney U p
Language barrier frequency	4.0 (4.0-5.0)	4.0 (4.0-5.0)	0.795
Interpreter confidence	2.5 (2.0-3.0)	3.0 (3.0-4.0)	0.118
Cultural barrier frequency	3.0 (3.0-4.0)	4.0 (3.0-4.0)	0.351
Cultural preparedness	4.0 (2.75-4.0)	3.5 (3.0-4.0)	0.884
Resource limitation frequency	2.0 (2.0-3.0)	3.0 (3.0-3.75)	0.149
Faculty/peer support	5.0 (4.0-5.0)	3.5 (3.0-4.0)	0.0005*
Pre-clinic training effectiveness	3.5 (3.0-4.0)	3.0 (2.0-3.0)	0.052^†^

Given the small subgroup sizes (pre-clinical n = 12, clinical n = 10), all pre-clinical/clinical comparisons should be interpreted as exploratory only. Clinical students reported higher interpreter confidence than pre-clinical students, though this difference was not significant (p = 0.118). Pre-clinical students rated pre-clinic training effectiveness more highly than clinical students (median 3.5 vs. 3.0, p = 0.052); given our sample size, this should not be interpreted as a near-significant trend but simply reported descriptively, and may reflect evolving expectations as students gain a clinical frame of reference.

Beyond the support-resource association reported above, additional exploratory Spearman correlations were examined among key study variables. Interpreter confidence was not significantly correlated with language barrier frequency (rho = -0.218, p = 0.329), suggesting that greater exposure to language-discordant encounters did not, on its own, translate into greater self-reported confidence using interpreters. Similarly, cultural preparedness was not significantly correlated with cultural barrier frequency (rho = 0.183, p = 0.416), indicating that students' self-assessed readiness to navigate cultural challenges was not closely tied to how often they actually encountered such challenges. Finally, the number of clinic sessions attended was not significantly correlated with interpreter confidence (rho = 0.302, p = 0.172), suggesting that repeated clinical exposure alone did not reliably build interpreter-use confidence over time. All correlations were exploratory, and given the small sample size, none should be interpreted as confirmatory.

Abstract Conceptualization and Active Experimentation stages - qualitative findings

Directed content analysis of 66 open-ended responses (mean three responses per participant) yielded four themes, each mapping to disruptions within Kolb's ELT cycle (Table 4). Inter-rater agreement prior to consensus was substantial (κ = 0.78).

**Table 4:** Directed content analysis themes mapped to Kolb’s ELT stages, with representative student quotations and proposed solutions ELT: Experiential Learning Theory

Theme	Kolb's ELT stage disrupted	Representative quotation	Proposed solution (student-generated)
Interpreter inaccessibility	Concrete Experience	"Half the time there was no interpreter and I had to rely on a family member who clearly didn't understand the medical terms."	On-demand telephonic interpretation; pre-session interpreter assignment protocol
Cultural and gender-concordant care gaps	Concrete Experience; Reflective Observation	"I didn't know how to approach a female patient who was uncomfortable with me, a male student, doing the physical exam."	Adaptive communication frameworks; gender-concordant team assignments when possible
Inadequate pre-clinic preparation	Abstract Conceptualization	"I knew theoretically about trauma-informed care but had no idea how to actually do it in a ten-minute encounter with no interpreter."	Structured pre-clinic briefings; simulation-based training; post-clinic debriefs
Structural workflow deficits	Active Experimentation	"Every time I came back, I had a completely different patient and no idea what had happened before."	Longitudinal patient assignments; structured handoff documentation; clear role delineation

Theme 1: Interpreter Inaccessibility (Concrete Experience Disruption)

Language barriers were described not merely as communication inconveniences but as fundamental disruptions to clinical reasoning. Students described forming incomplete histories, misinterpreting pain descriptions, and being unable to adequately screen for mental health symptoms in the absence of qualified interpretation. Reliance on patient family members, particularly children, was described as both clinically inadequate and ethically uncomfortable. The consistent absence of standardized interpreter protocols was identified as the most actionable structural deficit.

Theme 2: Cultural and Gender-Concordant Care Gaps (Concrete Experience and Reflective Observation Disruption)

Students described specific, recurring situations in which cultural unfamiliarity disrupted their clinical approach: difficulty completing physical examinations with male physicians examining female patients, uncertainty about culturally embedded explanatory models of illness, and discomfort navigating religious observance (e.g., fasting, prayer schedules) during medication counseling. Students expressed a desire not simply for more information about specific cultures but for adaptive communication frameworks applicable across diverse encounters.

Theme 3: Inadequate Pre-Clinic Preparation (Abstract Conceptualization Disruption)

Students consistently described a mismatch between their conceptual knowledge of refugee health challenges (acquired through coursework or reading) and their readiness to navigate those challenges in real encounters. Pre-clinic sessions were described as logistically focused (roles, room assignments) rather than educationally preparatory. Students proposed structured pre-clinic briefings, simulation-based practice with standardized patients portraying refugee scenarios, and post-clinic debriefing as mechanisms to strengthen the reflective and conceptual phases of the learning cycle.

Theme 4: Structural Workflow Deficits (Active Experimentation Disruption)

Students identified clinic-level structural issues that constrained their ability to apply learning across sessions: lack of patient continuity, unclear role delineation, no structured handoff between student cohorts, and inconsistent documentation practices. These deficits prevented Active Experimentation, the final ELT stage, from occurring, as students rarely returned to manage the same patient over time. Improved care continuity and role clarity were the most frequently proposed structural remedies.

## Discussion

This convergent parallel mixed-methods study examined medical student experiences in a student-run refugee health clinic through the lens of Kolb's ELT. By operationalizing ELT as both a survey framework and a qualitative analytic lens, we moved beyond simple description of barriers to identify where in the learning cycle those barriers are most disruptive and what targeted interventions might best address them.

Our central finding is that disruptions occur primarily at the Reflective Observation and Abstract Conceptualization stages of the ELT cycle, not at the Concrete Experience stage. Students are gaining abundant direct exposure to complex refugee health encounters. What is largely absent are the structured mechanisms debriefing, simulation, and mentored reflection that allow raw experience to mature into transferable competence. This distinction has direct programmatic implications: the solution is not more clinical hours but better educational scaffolding around the hours that already occur.

Language barriers were nearly universal and represent the most operationally critical disruption to effective learning and care. Our finding that 72.7% (16/22) of students reported inadequate formal training in interpreter use aligns with existing literature documenting systematic underpreparedness for cross-language clinical encounters among medical trainees [3,10]. Notably, interpreter confidence did not correlate with language barrier frequency (rho = -0.218, p = 0.329), consistent with prior work showing that unguided exposure to language-discordant encounters does not reliably build communication competence [10,15]. This supports the ELT prediction that Concrete Experience alone without Reflective Observation and Abstract Conceptualization does not produce competence growth. Structured interpreter training, including briefing and debriefing protocols, pacing strategies, and avoidance of child interpreters, has been shown to improve clinical communication outcomes and should be incorporated into student preparation [15,16].

Cultural challenges were nearly as prevalent as language barriers, yet students' self-assessed preparedness did not correlate with cultural barrier frequency, suggesting that self-assessed readiness may not reflect the applied skill demands of real clinical encounters, a gap consistent with Kolb's prediction that conceptual familiarity alone does not produce adaptive competence. This gap between perceived and actual readiness is well-documented in the cultural competency literature and reflects the limitations of knowledge-focused curricula [17-19]. The qualitative findings clarify the specific nature of this gap: students encountered real-time situations, gender-sensitive physical examinations, religiously contextualized medication counseling, and trauma-inflected histories for which conceptual frameworks alone were insufficient preparation. Simulation-based cultural training using standardized patient scenarios has demonstrated efficacy in improving adaptive communication skills and should be considered a high-priority curricular addition [20].

The significant negative association between perceived faculty and peer support and reported resource limitations (rho = -0.425, p = 0.049) is among the study's most theoretically generative findings. This finding suggests that the perception of resource constraints may not be simply a function of what is objectively available but could instead be associated with the quality of supervisory relationships. It is possible that supported students reframe constraints, limited supplies, time pressure, and incomplete data as manageable clinical puzzles rather than insurmountable barriers, though this study did not directly test this mechanism. This is broadly consistent with ELT's assertion that guided reflection transforms experience: faculty who scaffold students' thinking during encounters may help facilitate the Abstract Conceptualization that unsupported students appear less likely to achieve independently. It also extends prior work on the role of near-peer support in community clinic settings [16].

The pre-clinical/clinical student divergence in perceived support levels (p = 0.0005) merits attention. Pre-clinical students reported substantially higher support, which may reflect more intensive supervision in earlier training or, alternatively, lower expectations creating more satisfying encounters with existing support. Clinical students' lower support ratings may signal an important equity gap: as students progress to more independent practice, structured mentorship in community settings may paradoxically decrease. Ensuring that clinical-year students receive faculty-engaged supervision, not merely autonomous placement within refugee clinic settings, should be a priority for program directors.

Qualitatively, the structural workflow deficits identified by students highlight a frequently overlooked dimension of experiential learning quality. Patient continuity is not merely a patient safety concern; it is an educational prerequisite for Active Experimentation. Without returning to a patient, a student cannot test whether the adaptive strategy formulated in one encounter produced better outcomes. Designing longitudinal patient panels within student-run clinics, even informally, may help support the final stage of Kolb's cycle.

Implications for medical education

Based on the findings and their theoretical grounding in ELT, we propose four targeted interventions organized by the Kolb's stage they are designed to activate. These interventions were not implemented or evaluated within the present study; they are proposed strategies for future implementation and evaluation, informed by our findings and by existing educational literature, rather than evidence-based conclusions arising directly from our data.

For Reflective Observation, implement structured post-encounter debriefing at every clinic session. Debriefing guided by ELT prompts - What happened? What would you do differently? What principle does this illustrate? - activates the reflective stage that currently occurs inconsistently or not at all [16,21,22].

For Abstract Conceptualization, develop pre-clinic simulation modules using standardized patients in language-discordant and culturally complex scenarios. Simulation provides a low-stakes environment for conceptual principle formation that translates to better real-encounter performance.

For Active Experimentation, assign longitudinal patient panels to student volunteer cohorts, enabling follow-up across sessions. Even one planned return visit substantially increases the experiential learning depth available from each patient relationship.

For Concrete Experience (optimizing it), establish standardized interpreter protocols - including identification of available live, telephonic, and video resources - to ensure that language-discordant encounters are clinically productive rather than compromised. Provide students with an adaptive cultural communication framework (not a culture-specific fact sheet) in their orientation materials.

Limitations

This study has several limitations. The single-site design and small sample size (n = 22) limit generalizability and preclude confirmatory statistical inference; findings should be interpreted as hypothesis-generating. The small sample also limits statistical power, increasing the likelihood of both false-negative and false-positive results in our correlation and comparison analyses, and precludes multivariable or mediation analyses that could clarify the mechanisms underlying observed associations. The 22-item survey instrument was developed de novo and, while informed by literature review, expert review, and cognitive pilot testing, did not undergo formal psychometric evaluation, including content or construct validity assessment, internal consistency testing (e.g., Cronbach's alpha), or factor analysis. This absence of psychometric evaluation is a substantial limitation that reduces confidence in the reliability and construct validity of the Likert-scale measures used, and findings based on this instrument should be interpreted with corresponding caution.

Selection bias is possible, as students who volunteered for the clinic and chose to complete the survey may differ systematically from non-respondents or non-volunteers in motivation, cultural interest, or prior experience, potentially inflating reported levels of preparedness or perceived support relative to the broader student population; non-response among eligible students may further limit representativeness. Additionally, because barrier frequency, preparedness, and perceived support were all measured via the same self-report survey instrument, common method bias may have inflated the observed associations between these variables (e.g., the correlation between perceived support and resource limitations). Finally, given the single-site design and the specific linguistic and cultural composition of this clinic's patient population, the transferability of these findings to other student-run clinic models or refugee populations with different characteristics is uncertain and should be assessed in future multi-site research. The cross-sectional design captures a single point in time and cannot establish causal or longitudinal relationships between perceived support, preparedness, and outcomes. Self-reported measures of preparedness and confidence are subject to social desirability and recall bias. Finally, because the clinic serves a specific refugee population in one geographic region, findings may not generalize to other student-run clinic models or patient populations with different cultural and linguistic compositions.

## Conclusions

Medical students in student-run refugee health clinics encounter substantial, specific, and structurally addressable learning disruptions. When analyzed through Kolb's ELT, our data suggest these disruptions may be concentrated primarily in the Reflective Observation and Abstract Conceptualization stages, rather than in direct clinical exposure, which our findings indicate is already occurring at high volume; this interpretation should be considered hypothesis-generating given the study's exploratory design. The implication is clear: programs should invest in the educational infrastructure surrounding refugee clinic experiences, not simply in increasing the number of encounters. Structured debriefing, simulation-based preparation, enhanced faculty mentorship, and longitudinal patient continuity are theoretically grounded, practically achievable interventions that this study identifies as high-priority targets. As refugee and immigrant populations continue to grow in the United States, the ability to prepare medical students for equitable, culturally competent care is not a peripheral educational aspiration; it is a core competency demand that student-run clinic programs are uniquely positioned to meet.

## Appendices

Appendix A

Survey Instrument (22 Items)

By continuing, you confirm that: You are at least 18 years old You have read and understood the consent information You voluntarily agree to participate

Do you consent to participate in this research study? Yes, I agree to participate No, I do not agree to participate (→ Redirect to end of form if possible)

Section 1: Demographics

1. What is your age? Under 25 25-30 31-35 Over 35

2. What is your gender? Male Female Non-binary/Other Prefer not to say

3. What is your current level of medical training? Pre-clinical (1st or 2nd year medical student) Clinical (3rd or 4th year medical student) Other (please specify): ____________

4. How many times have you participated in the refugee health clinic? 1-3 times 4-6 times More than 6 times

5. Do you speak a language commonly used by the refugee populations you serve? Yes No Somewhat (basic phrases only)

Section 2: Challenges Faced

Language and Communication Barriers

6. How often do you encounter language barriers when treating refugee patients? Never Rarely Sometimes Often Always

7. Rate your confidence in using interpreters during patient encounters. 1 (Not confident at all) 2 (Slightly confident) 3 (Neutral) 4 (Confident) 5 (Very confident)

8. Do you feel you have adequate training to address language barriers in clinical settings? Yes No

Cultural Competence 

9. How often do cultural differences create challenges in patient care? Never Rarely Sometimes Often Always

10. Have you ever felt unsure about addressing cultural or religious sensitivities with refugee patients? Yes No

11. How prepared do you feel to provide culturally competent care? 1 (Not prepared at all) 2 (Slightly prepared) 3 (Neutral) 4 (Prepared) 5 (Very prepared)

Resource Availability

12. How often do you feel limited by the lack of medical tools or devices during clinic sessions? Never Rarely Sometimes Often Always

13. What specific resources do you feel are most needed at the refugee health clinic? (Open-ended response)

Emotional and Psychological Challenges

14. Have you ever felt emotionally overwhelmed while treating refugee patients due to their trauma or health conditions? Yes No

15. How well do you feel supported by faculty or peers in coping with these challenges? 1 (Not supported at all) 2 (Slightly supported) 3 (Neutral) 4 (Supported) 5 (Very supported)

Time and Training 

16. Do you feel you have enough time during clinic sessions to provide comprehensive care? Yes No

17. Rate the effectiveness of the pre-clinic training sessions in preparing you to address refugee health needs. 1 (Not effective at all) 2 (Slightly effective) 3 (Neutral) 4 (Effective) 5 (Very effective)

18. What additional training or resources would improve your ability to deliver care in refugee clinics? (Open-ended response)

Section 3: Reflections and Solutions

19. Describe a challenging experience you had while treating a refugee patient and how you handled it. (Open-ended response)

20. What do you think is the single greatest barrier medical students face in refugee health clinics? (Open-ended response)

21. What solutions would you recommend to address the challenges you've experienced? (Open-ended response)

Section 4: Additional Comments

22. Do you have any additional comments or suggestions about improving the experience for medical students in refugee health clinics? (Open-ended response)
